# Effects of Cryolipolysis on Abdominal Adiposity

**DOI:** 10.1155/2016/6052194

**Published:** 2016-11-08

**Authors:** Patricia Froes Meyer, Rodrigo Marcel Valentim da Silva, Glenda Oliveira, Maely Azevedo da Silva Tavares, Melyssa Lima Medeiros, Camila Procopio Andrada, Luis Gonzaga de Araujo Neto

**Affiliations:** Potiguar University (UnP), Laureate International Universities, 59054-180 Natal, RN, Brazil

## Abstract

Cryolipolysis is a noninvasive technique of localized fat reduction. Controlled cold exposure is performed in the selective destruction of fat cells. The aim of this study was to investigate the effects of cryolipolysis on adipocytes elimination through histological and sonographic analyses. This study reports the case of a 46-year-old female patient, with complaint of localized abdominal fat and in the preoperative period of abdominoplasty. The patient was submitted to a single 60-minute application of cryolipolysis, temperature of −5°C, on the hypogastrium area, 5 cm below the umbilicus. To study the effects of this treatment, ultrasound images taken before the session and 7, 15, 30, and 45 days after the therapy were analysed. After the abdominoplasty, parts of the treated and the untreated withdrawn abdominal tissues were evaluated macro- and microscopically. In ultrasound images, as well as in macroscopic and histological analyses, significant adipocytes destruction was detected, with consequent fat layer reduction and integrity of areas that were adjacent to the treated tissue. The presence of fibrosis observed during therapy and acknowledged through performed analyses encourages further studies to clarify such finding.

## 1. Introduction

Excessive localized fat and body weight as result from increased caloric intake in detriment of energy demand promote an important public health issue, characterized by the dissemination of diseases such as hypertension, diabetes mellitus type II, cardiovascular risk diseases such as atherosclerosis, dyslipidemia, and acute and chronic inflammatory processes, among others, besides favoring great physical and aesthetic dissatisfaction [1, 2].

Fat removal and body reshaping are increasingly popular cosmetic procedures. Currently, liposuction is the most common and effective procedure for body contouring. Given the invasive nature of liposuction, and its inherent risks, there has been continuous research for the development of noninvasive methods. Several noninvasive techniques have been experimented, such as laser applications, ultrasound, radiofrequency, and infrared light, with variable demonstration of scientific efficacy [3].

The development of a noninvasive method that operates in the reduction of the fat layer called “cryolipolysis” has been employed for the selective destruction of fat cells. This nonsurgical approach uses controlled cooling to decrease subcutaneous fat without damaging surrounding tissues [4]. The equipment maintains the previously set temperature below 0°C throughout the application, by means of incorporated sensors within the cooling plates located on each side of the applicator [5]. Thus, the cold induces an inflammation response which causes the adipocyte programmed death (apoptosis) and, thereby, gradually lessens the fat layer [3–6]. Cryolipolysis effects are not immediate; however, statistically significant reduction may be obtained within approximately two months from application [7].

Apoptosis of fat cells (adipocytes) is initiated when these cells are cooled to the temperature of −1°C. However, the destruction of adipocytes does not affect serum lipid levels or liver function tests significantly. In spite of being a new technology without a fully understood mechanism of action, promising results have been confirmed in studies; therefore, it seems to be an excellent alternative for localized fat reduction without major adverse effects [7–9]. This case report was conducted in order to validate the effects of this therapy in eliminating abdominal fat cells, through histological and ultrasound examinations.

## 2. Material and Methods

The research was characterized as a case study in which the subject was a 46-year-old female participant. The absence of contraindications to the use of cryolipolysis equipment (cold hypersensitivity and autoimmune diseases), the presence of localized abdominal fat, and being in a preoperative period of abdominoplasty surgery were the observed aspects, which were considered for the acceptance of this subject for this study. Among the applied exclusion criteria, it was determined that no individuals with contraindications for the use of cryolipolysis equipment or voluntary withdrawal would be shortlisted.

The research was conducted in the city of Natal, RN, Brazil, in the premises of the Potiguar University Integrated Health Clinic (CIS), and approved (Registered under number 1,249,981) by the Potiguar University Ethics Committee (CEP), Natal, RN, Brazil.

A high-frequency ultrasound device (Medson, Korea, 5–13 MHz), a nonprofessional camera (Sony, Cyber-shot DSC-W350, USA), a tape-measure (Fiber Glass Tape, China), a scale (Accumed-Glicomed, Rio de Janeiro, Brazil), an optical binocular microscope apparatus (OLYMPUS, CX31 model, USA), and a cryolipolysis device (Galeno, South Korea) were used.

### 2.1. Procedures

Prior to treatment application, the volunteer signed the informed consent form. Then, she was submitted to the PAFAL protocol, validated by Meyer et al. [10], which addresses the following topics: identity, anamnesis, lifestyle habits, physical examination, measurement, and tests concerning weight, height, BMI, skinfolds, and abdominal circumference. For latter accomplishing, the patient was placed in the standing position and the tape was positioned 5 cm below the umbilicus, parallel to the floor.

Subsequently, the patient underwent ultrasound examination, which was performed by a medical specialist at the Potiguar Image Service clinic (SIP). The examination was performed on the infraumbilical region, in a 10 cm^2^ pen marked area, just below the umbilicus, while the volunteer was positioned in dorsal decubitus position. Sonography was performed in linear probe multifrequency mode with probe of 5–13 MHz, 5.0 cm penetration, engaging the nozzle longitudinally and transversely with light pressure, for data obtention on inflammation, thickness of the fat layer in cm, density, and fibrosis. Sonography was performed in 5 distinct periods: before application and 7 days, 15 days, 30 days, and 45 days after the procedure.

**Figure 1:**
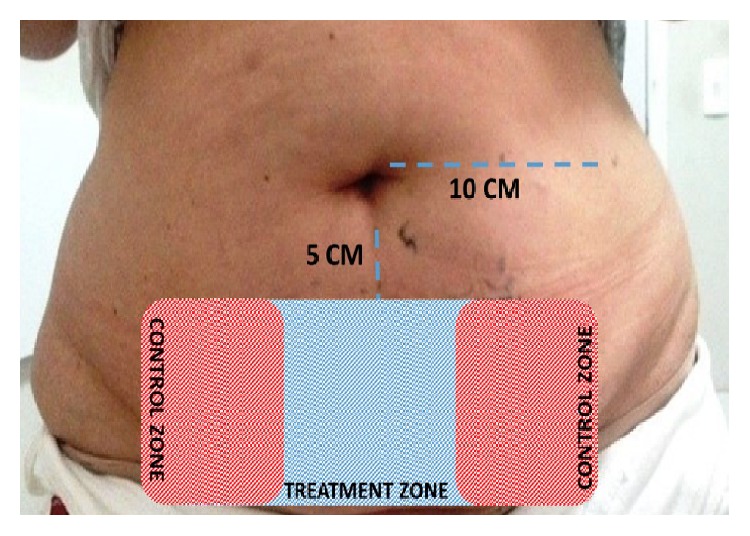
Marked area for treatment (treatment zone and control zone). Each area had 10 cm × 10 cm, with total surface 30 × 10 cm.

To perform the treatment, demarcation of the area was initially made with the use of a gentian violet ink marker on infraumbilical region, 5 cm below the umbilicus toward the symphysis pubis and 10 cm to the side, toward the waist. The treated area was in the lower central part of the abdomen as shown in Figure 1.

The left and right side area received a same-size marking and served as control. The participant was submitted to a single application session of cryolipolysis, for 60 minutes, with a suction pressure of 60 kPa and temperature of −5°C. The application session was performed with volunteer in the dorsal decubitus position with a 45-degree stretcher inclination. The patient was reevaluated in terms of overall weight and body perimeter with the ultrasound 7, 15, 30, and 45 days after the first application.

**Figure 2:**
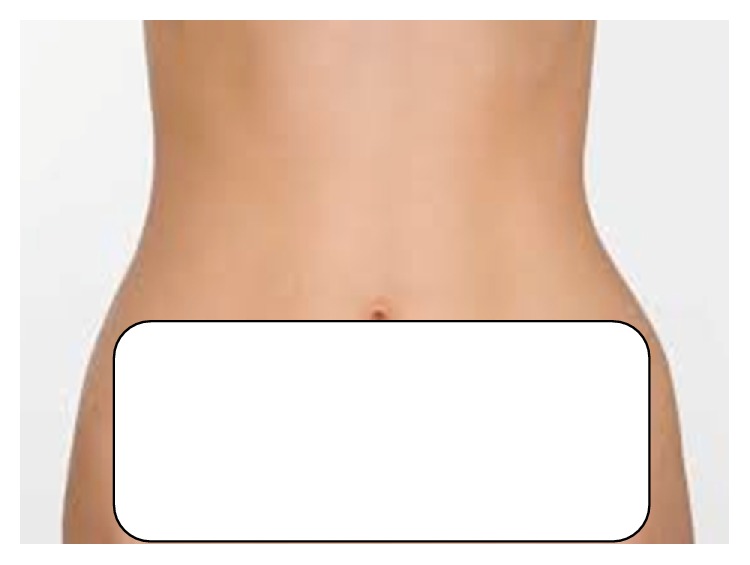
Marked area for abdominoplasty.

Forty-five days after the cryolipolysis treatment session, the patient was submitted to an abdominoplasty. In preparation for surgery, the two pretreated areas were marked again. With clear identification (Figure 2), the marked areas were cut by the surgeon inside the pavilion.

During the abdominoplasty procedure, abdominal skin fragments from the pretreated area were separated for histopathological analysis. The pieces were fixed in 10% formaldehyde for 48 hours, cleaved, and processed according to pathology laboratory routine protocols (dehydration, diaphonization, imbibition in paraffin, and inclusion). The resulting paraffin blocks were cut with a rotary microtome of 5 *μ*m thickness. Slices were obtained and stained with the hematoxylin and eosin technique (HE). For microscopic analysis, the slices were examined under an optical binocular microscope (OLYMPUS, model CX31, USA) with attached camera. The examination was performed in a tabulated and blind fashion, using parameters for inflammatory/reparative processes. The microscopic field photos were obtained in two magnification settings (100x and 400x).

### 2.2. Data Analysis

Qualitative data were described based on medical reports (descriptive analysis of ultrasound images) and based on the pathologist reports (descriptive analysis of histological images). Data collection and correlation were presented in tables and figures.

## 3. Results

From the cryolipolysis application day and until the conclusion of abdominoplasty surgery, the patient did not change her diet. The variables measured before and 45 days after treatment are reported in Table 1.

There was a mean reduction of 3.53 cm in the abdominal circumference. Regarding weight, 70 kg before treatment and 69.7 kg after treatment were recorded, with a slight decrease of 300 g in overall body weight.

### 3.1. Ultrasound Results

Ultrasound data obtained before and after cryolipolysis application session were registered in Table 2.

**Figure 3:**
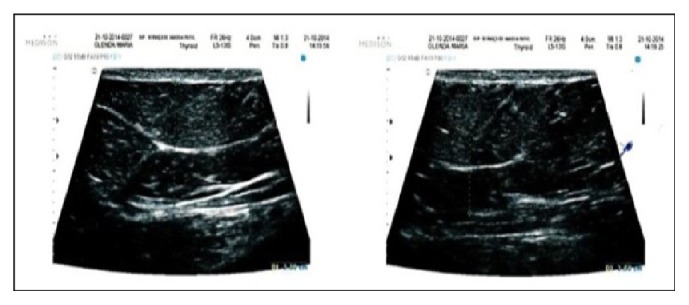
Sonography before treatment.

**Figure 4:**
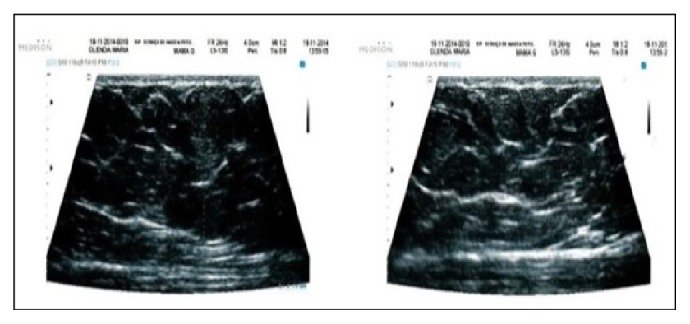
Sonography, 7 days after treatment.

**Figure 5:**
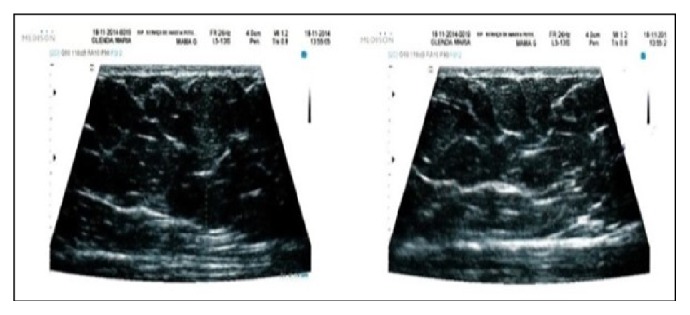
Sonography, 15 days after treatment.

**Figure 6:**
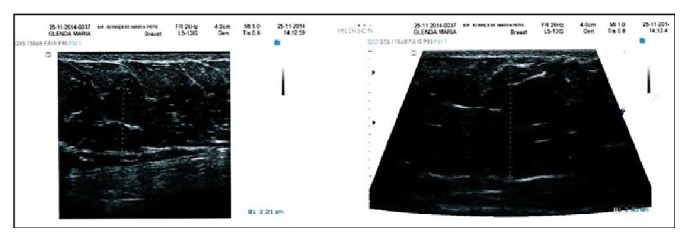
Sonography, 30 days after treatment.

**Figure 7:**
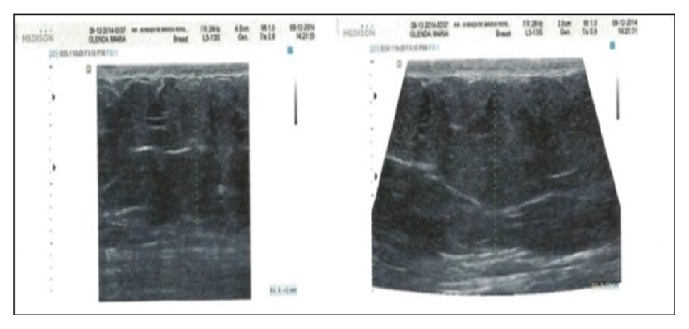
Sonography, 45 days after treatment.

The qualitative analysis performed by the sonographer is demonstrated in Figures 3, 4, 5, 6, and 7.

The results obtained with the ultrasound demonstrate a decreasing in the fat layer thickness averaged 1.46 cm after the treatment. It was also possible to verify the presence of fibrosis before treatment, increasing the fibrous septa on an average of 0.12 cm after the therapy.

After 7 days, a mild inflammation process and sharp fibroses were identified. During this period, an average decrease of 1.08 cm on the adipose layer thickness and an average thinning of the fibrous septa of 0.04 cm were registered.

Fifteen days after treatment, there was no identified inflammation. The ultrasound measurement showed an average decrease of 0.02 cm in the fat layer thickness and an average of 0.60 cm increase in the fibrous septa thickness.

A significant reduction in local tissue fibrosis was observed 30 days after treatment. In this phase, the fat layer thinning presented an average of 0.16 cm and the thinning of the fibrous septa, 0.50 cm.

A discrete return of fibrous tissue was observed 45 days after treatment. There was an average of decrease 0.20 cm in the fat layer thickness at this stage and an increase of 0.06 cm in the fibrous septa thickness.

Table 3 demonstrates sonography findings, which demonstrate absence of inflammatory process before treatment and inflammatory peak 7 days after treatment. Fibrosis was present and detected prior to treatment, which may be a consequence of a liposuction procedure the volunteer underwent 10 years ago. Fifteen days after treatment, the fibrosis increased in thickness, at 30 days no fibrosis was observed, and 45 days after the therapy new fibrosis arose.

### 3.2. Macroscopic and Microscopic Analyses Results

**Figure 8:**
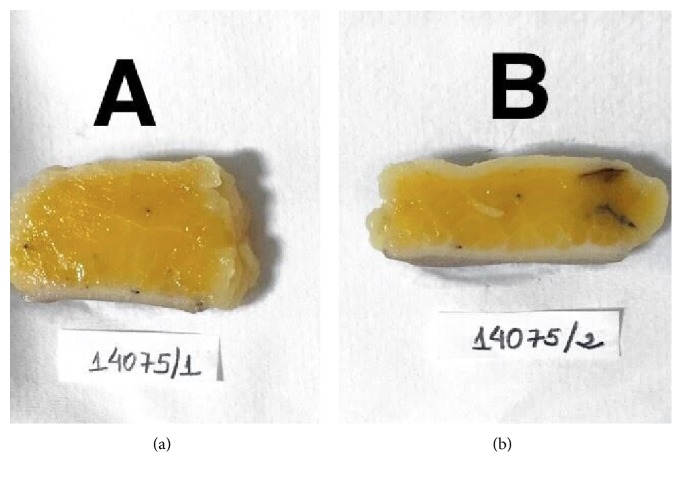
Macroscopic analysis of the material collected during the abdominoplasty surgery. (a) Untreated side with weight of 546 g and size 15 × 10 cm. (b) Treated side weight of 508 g and size 15 × 10 cm.

Figure 8 demonstrates the material collected by the plastic surgeon from the treated and untreated area, cut, and prepared for macro- and microscopic analysis. The sample, collected during the abdominoplasty surgery, (removed area of untreated tissue) had weight of 546 g and 15 × 10 cm in length. The tissue removed from the treated area had weight of 508 g and 15 × 10 cm in length, with 38 g less than the tissues of the untreated area.

Three tissue samples, corresponding to the medial (treated) and lateral (untreated) areas, were extracted and utilized for the histological records. Macroscopically visualizing the removed tissue, it is clear that the first one, on the left side of the photo (control), is visually thicker than the right side sample (with cryolipolysis).

**Figure 9:**
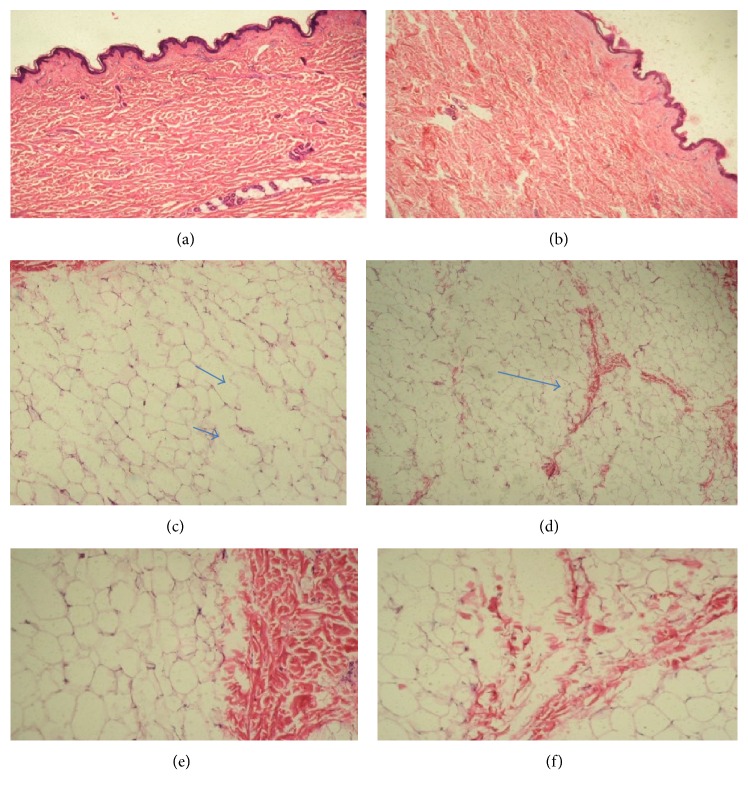
Microscopic analysis of the material collected during the abdominoplasty. Longitudinal micrograph HE 100x (a) untreated region and (b) treated region. Longitudinal micrograph HE 100x (c, d) adipocytes membranes destruction and fibrosis in the midst of the adipose tissue. (e, f) Longitudinal micrograph HE 400x treated area with preserved adjacent tissue and fibrosis in the midst of the adipose tissue.

Concerning the histological records, normal appearance was observed in both treated and untreated adipose tissue and in adjacent tissues (Figures 9(a) and 9(b)).

In the Figure 9(e), the integrity of the surrounding areas (untreated) is noticed. It was also possible to visualize areas with localized infiltrates and macrophages, suggesting an inflammatory condition (Figure 9(f)). Fibrosis in the middle of adipose tissue and adipocytes lysis may be detected in the epidermis of the treated group, indicating lack of continuity in the tissue morphology and absence of adipocytes in specific areas (Figures 9(c) and 9(d)).

## 4. Discussion

This study indicated reduction of 3.53 cm in the average abdominal circumference perimeter, which was also found in Ferraro et al.'s study [11], in which 50 patients with abdominal localized fat were selected and 14 underwent cryolipolysis treatment. In Ferraro et al.'s study, a significant circumference reduction of the treated area was verified, with an average decrease of 4.45 cm by end of therapy period.

In this study, subjects were submitted to one single cryolipolysis application. Zelickson et al. [4] conducted a study with 42 patients who underwent a single application of cryolipolysis in the thigh area. The results showed significant reduction of the fat layer through circumference measurement and ultrasound images 16 weeks after treatment.

In the present study, the volunteer patient did not change her diet and kept body weight constant through treatment. In his work, Ferraro et al. [11] also found that all patients kept body weight constant, strongly suggesting that reduction in fat thickness was due to local treatment.

Seven days after treatment, an inflammatory process peak was identified, followed by its reduction from the 15th until the 45th day after application. A study associated with histological analysis carried out by Boey and Wasilenchuk [12] showed increasing inflammatory response, which reached its peak 30 days after treatment, along with dense infiltrate of inflammatory cells and reduction in adipocytes size followed by similar decreasing response after 60 and 120 days, with reduced adipocytes size and infiltrates decrease. Avram and Harry [3], in their essay, performed several histological analyses in a variety of time periods after exposure to the cold through cryolipolysis and results showed an inflammatory peak around 14 days after treatment and turned out well after 30 days, with the visually decreased inflammatory response and volume of fat cells. The findings of Avram and Harry [3] coincide with this study in relation to the peak inflammation and increased of fibrous septa thickness. In the present study, presence of fibrosis in the middle of adipose tissue was verified through ultrasound images before treatment and 7, 15, and 45 days later, with the most significant fibrous thickness in the 15th day.

Stevens [13] observed skin firmness in patients having flaccidness and attributed results to the cryolipolysis therapy, and even those who achieved significant fat volume reduction did not show skin flaccidity. Instead, 4 months after treatment, the firm skin adhered well to its new body contours. The mechanism through which cryolipolysis induces skin firmness is not well understood but may result from stimulated collagen production, new elastin formation, fibrosis, or tissue compression. According to Carruthers et al. [14] cryolipolysis may stimulate neocollagenesis by stretching of the fibroblasts. Except for the conformable surface applicator, most cryolipolysis treatments are delivered using vacuum applicators. The vacuum suction that pulls the tissue bulge into the treatment cup may provide mild stretching to the skin and contribute to neocollagenesis.

Histological analysis demonstrated adipocytes membranes destruction and fibrosis. A study by Zelickson et al. [5], with pigs as subjects and aiming cryolipolysis fat removal confirmed through histological analysis adipocytes loss of mononuclear lipid cells emergence, inflammatory convey, and local thickening of fibrous septa. Coleman et al.'s work [15] observed the existence of reduction in the fat layer through ultrasound images after a single treatment. In the study by Stevens and Bachelor [16], with 40 patients as subjects, ultrasound data indicated significant fat layer reduction.

The results related to adjacent regions integrity are consistent with those reported in literature. Coleman et al. [15] and Zelickson et al. [5] perceived the integrity of treatment adjacent areas, finding no clinical or histological evidence of skin lesions and no scar.

Therefore, the security of cryolipolysis therapy and its effectiveness has been widely quoted, corroborating with the findings of this study. It is important to emphasize that the vast majority of conducted and published studies use equipment with CIF (cooling intensity factor), but most of the equipment used in Brazil and other countries in Latin America do not have this type of system which securely displays applied temperature settings [17–19]. In this sense, the present study is relevant, even as a case study, as it analyses, through different methodologies, the clinical picture evolution of a patient undergoing cryolipolysis treatment. Further studies with more volunteers are suggested.

In conclusion, in both histology and ultrasound images analyses, it was possible to highlight important destruction of fat cells with consequent reduction in the fat layer, along with integrity maintenance of treatment adjacent areas. The presence of fibrosis observed throughout therapy encourages further studies to clarify this finding.

## Figures and Tables

**Table 1:** Anthropometric data of the patient.

Evaluation of measures and weight	Before (cm)	7 days later (cm)	15 days later (cm)	30 days later (cm)	45 days later (cm)
Abdominal circumference 5 cm above the umbilicus scar	99.5	98.2	99.4	97	97
Abdominal circumference 5 cm below the umbilicus scar	107	105.5	104	103.7	103.8
Umbilicus scar	109	108.3	107.7	103.5	104.1
Weight (kg)	70 kg	70 kg	69.5 kg	68.5 kg	69.7 kg

**Table 2:** Thickness measurement of the fat layer and fibrous septa based on ultrasound data.

Measurements	Before	7 days later	15 days later	30 days later	45 days later
Adipose layer thickness (average)	3.67 cm	2.59 cm	2.57 cm	2.41 cm	2.21 cm
Fibrous septa thickness (average)	0.10 cm	0.06 cm	0.66 cm	0.16 cm	0.22 cm

**Table 3:** Description of the sonography results regarding the inflammatory process and fibrosis presence.

	Before	7 days later	15 days later	30 days later	45 days later
Inflammatory process	Absent	++	+	+	+
Fibrosis	++	++	+++	Absent	+

Absent: inexistent; +: light; ++: moderate; +++: intense; source: sonography data.
